# Exploring the genetic basis of Newcastle disease virus in chickens: a comprehensive review

**DOI:** 10.3389/fimmu.2025.1614794

**Published:** 2025-06-27

**Authors:** Haile Berihulay, Wei Luo, Ainong Lao, Jian Ji, Manshan Cai, Dingming Shu, Chenglong Luo

**Affiliations:** ^1^ State Key Laboratory of Swine and Poultry Breeding Industry Guangdong Key Laboratory of Animal Breeding and Nutrition Institute of Animal Science, Guangdong Academy of Agricultural Sciences, Guangzhou, China; ^2^ College of Natural and Computational Science, Aksum University, Aksum, Tigray, Ethiopia

**Keywords:** chicken, immune response, molecular structure, NDV, pathogenesis, genetic resistance

## Abstract

Newcastle disease (ND) is one of the most serious viral diseases affecting chickens and is caused by Newcastle disease virus (NDV), an avian paramyxovirus serotype-1. The virus contains five structural proteins and two nonstructural proteins that interact with the host proteins involved in viral infection and host antiviral responses. Currently, several NDV strains have been molecularly characterized; however, a comprehensive overview of NDV in chickens that addresses recent findings is lacking. This review summarizes the current report of the molecular structure of NDV, including candidate genes and genomic regions, virulence and route of infection, mechanisms of resistance, host immune response, disease resistance mechanisms and effects of NDV on chicken immune performance. Therefore, this review can be used by researchers seeking a comprehensive understanding that can be applied in future breeding programs aimed at enhancing disease resistance.

## 1 Introduction

Newcastle disease (ND) is a devastating and highly contagious disease that causes significant economic losses in the poultry industry by generating digestive, neurological, and respiratory symptoms (1). ND is caused by avian orthoavulavirus 1 (AOAV-1), commonly referred to as Newcastle disease virus (NDV) (2). The virus belongs to the order Mononegavirales, family Paramyxoviridae, and genus Orthoavulavirus (3). The genome is composed of a negative-sense, single-stranded RNA of approximately 15.2 kb coding for six structural proteins: nucleocapsid protein (NP), phosphoprotein (P), matrix protein (M), fusion protein (F), hemagglutinin-neuraminidase (HN), and large polymerase protein (L), arranged in the order of 3’-NP-P-M-F-HN-L-5’ (4). Variations in the hemagglutinin-neuraminidase (HN) and fusion (F) proteins lead to differences in the strains, affecting the virus’s ability to infect and cause disease (2). NDV strains vary in virulence and are categorized into three pathotypes based on their pathogenicity in chickens: lentogenic (low or avirulent), mesogenic (moderate virulence), and velogenic (high virulence) (5). Among the various strains of NDV, high levels (velogenic) strains typically cause severe neurological and respiratory symptoms, such as tremors, incoordination, paralysis, and sudden death (5). The virus can infect multiple tissues, including respiratory, digestive, and nervous systems, leading to destructive effects on poultry farms. The high morbidity and mortality rates of velogenic NDV strains are often associated with significant financial losses due to the mass culling of infected flocks, decreased production, and the cost of implementing control measures to prevent further spread (6). This genetic diversity of NDV strains complicates disease management and mitigation, emphasizing the need for a deeper understanding of the virus (7). The high genetic variability of NDV, virulent strains that may evade vaccine protection are more likely to emerge, potentially leading to larger epidemics with significant economic losses. Therefore, monitoring circulating NDV genotypes is crucial to reducing the impact of ND. Recently, various diagnostic strategies have been introduced, with continuous surveillance and strain characterization being key to maintaining the effectiveness of diagnostic tools and vaccines as NDV evolves (8).

In recent years, several approaches such as genome-wide association studies (GWAS), gene editing, deep sequencing, and transcriptomics have been developed to understand the viral evolution, host-pathogen interactions, vaccine development, and resistance mechanisms in chickens (6). These advancements allow for the identification of genetic markers linked to disease resistance across different chicken breeds by analyzing immune responses to NDV infection, which involves both innate and adaptive immunity (5). The involvement of genes and signaling pathways in NDV resistance highlights the potential for genetic selection based on these markers, which could significantly enhance the immune response in chickens (9).

Considering the expanding global livestock and poultry industry and the increasing volume of international trade, effective ND prevention strategies are particularly important. These strategies include strict biosecurity measures, rigorous vaccination programs, active surveillance, and proper disposal of infected carcasses (10). As the poultry industry evolves, the implications of ND extend beyond immediate health concerns to broader economic impacts. Frequent outbreaks also threaten animal health and disrupt trade relationships and markets. Therefore, understanding the persistence of NDV strains highlights the necessity for continuous molecular characterization of the virus, provision of updated information, and ongoing research into more effective vaccines and innovative control measures (11). Globalization facilitates the rapid movement of goods and animals, increasing the risk of disease spread and necessitating effective surveillance and response strategies to mitigate potential outbreaks of new diseases (11). Addressing these challenges is important for protecting public health and ensuring the sustainability of the poultry industry in an interconnected world. This approach not only addresses existing health issues but also supports long-term strategies for managing ND outbreaks globally. This review will concentrate on the current research findings on NDV, including insights into its molecular characteristics, genomic structure, pathogenicity, and mechanisms of infection and disease resistance. Additionally, we will highlight host genomics and innovative tools for NDV detection, including viral diagnostic methods and the impact of NDV.

## 2 Molecular characterization of NDV

Molecular characterization of NDV refers to the assessment of the virus’s genetic material, protein constituents, and molecular mechanisms that facilitate its replication, pathogenesis, and interaction with host cells. Understanding these molecular characteristics is critical for the development of vaccines, effective disease control strategies, precise diagnostic tools, and therapeutic approaches (12). More interestingly, molecular characterization encompasses several important aspects, including pathogenesis, mechanisms of infection, structures, host genomic interactions, and diagnostic approaches. The following sections provide a detailed overview of these aspects.

### 2.1 NDV classification

Based on phylogenetic analyses, NDV strains are classified into two primary classes: Class I, which generally includes avirulent strains primarily found in wild birds, and Class II, which encompasses both virulent and avirulent strains that affect various avian species, including domestic chickens (13). Within Class II, over 21 genotypes (I–XXI) have been identified, with notable variations in virulence that necessitate a classification system based on the severity of the diseases they cause (13). Similarly, Jia et al. (14) reported that class I NDVs were the most dominant population, with the identification of sub-genotype 1.1.2-accumulating mutations that potentially increase virulence in central China. A comprehensive study of 1,065 farms in 18 provinces revealed that the prevalence of NDV in chickens gradually declined from 1.49% in 2019 to 0.44% in 2022 at the bird level in China (1). A previous report from the Moscow region of Russia showed approximately 15 outbreaks of NDV in August 2022, with an additional three cases documented in 2023 (15). Qiu et al. (16) evaluated the biological properties of genotype VII, particularly its pathogenicity and viral replication patterns, following different inoculation routes, including intranasal, intraocular, and cloacal infections. Their findings revealed that intravenous injection induced higher morbidity and mortality, whereas intranasal, intraocular, and cloacal routes led to slower disease progression and less severe clinical symptoms than other routes of administration. Similarly, Sarika et al. (17) highlighted an important genetic relation between velogenic (highly virulent) strains of NDV and the XIII.2.2 sub-genotype circulating in India, suggesting that the XIII.2.2 sub-genotype may be associated with high pathogenicity.

To date, about half of the NDV class II genotypes, including I, IV, V, VI, VII, XI, XIII, XIV, XVII, XVIII, and XXI, with genotype VII being the most prevalent, have been reported in Africa (18). A study by Amoia et al. (19) revealed the high genetic diversity of virulent NDV strains circulating in almost all African countries, with genotypes II, IV, V, and VII being the most prevalent in East Africa, including Sudan, Somalia, Seychelles, Kenya, Uganda, South Sudan, Djibouti, Burundi, Ethiopia, Tanzania, Eritrea, Mauritius, Rwanda, and Comoros. Genotype VII has emerged as a particularly virulent variant and can be further subdivided into three subgenotypes: VII.1.1, VII.1.2, and VII.2 (5). Sub-genotypes VII.1.1 and VII.2 circulate predominantly in southern Africa, including Angola, Malawi, Eswatini, Lesotho, Zimbabwe, Zambia, Namibia, Botswana, Mozambique, and South Africa, and are predominant in northern Africa, particularly in Tunisia, Morocco, Western Sahara, Algeria, Egypt, and Libya (7, 20). Furthermore, these sub-genotypes (VII.1.1 and VII.2) were involved in the fourth NDV panzootic during the 1990s in Europe, Asia, and the Middle East, whereas VII.2 was first identified in Indonesia (21). Genotype XIII has also emerged, affecting regions such as southern, western, and central Asia and Africa (21). Similarly, genotype XVII is the most geographically dispersed genotype in Central Africa (Equatorial Guinea, Chad, São Tomé and Príncipe, Central African Republic, Democratic Republic of Congo, Cameroon, Gabon, and Congo-Brazzaville) and West Africa (Nigeria, Niger, Burkina Faso, Benin, and Mali) (19). Molecular analyses of the fusion (F) protein-encoding gene have revealed the presence of velogenic genotypes, including genotype VII, in Egypt and Saudi Arabia (22, 23).

Phylogenetic and network analyses have provided insights into the evolutionary dynamics and transmission patterns of NDV in chicken populations (24). A study using phylogenetic tree analysis revealed that NDV belongs to genotype VII, ranging from 99.7% to 98.5% with isolates from Bangladesh, Iran, and India (25). Furthermore, a recent investigation in Tanzania reported a 25.23% positivity rate for NDV in backyard chickens, emphasizing the ongoing issue in live bird markets (21). Bahoussi et al. (26) identified potential recombination events among NDV strains circulating in different provinces of China, involving strains from various genotypes and host species (chickens, ducks, and swine). Furthermore, phylogenetic analysis has identified two distinct major groups: Group I (GI), which includes a single genotype (Ib), and Group II (GII), which consists of eight genotypes (I, II, III, VI, VII, VIII, IX, and XII) (26. Notably, the Ib genotype is predominant in China, accounting for 34% of cases, with a strong concentration in the southern and eastern regions. Following Ib, genotypes VII and VI are also prevalent, representing 24% and 22% of cases, respectively (26). Sultan et al. (27) assessed a genotype VII-matched vaccine in commercial layers and found that the combination of a recombinant, genotype-matched inactivated vaccine and a live attenuated vaccine effectively reduced virus shedding and enhanced egg production in layers challenged with a velogenic genotype VII virus under field conditions. Overall, in this review, we have assessed and summarized the various types of NDV strains, their prevalence, and regional distribution (**Table 1**).

**Table 1:** The Newcastle disease virus genotype, genetic diversity and geographic distribution across different regions.

Genotype	Classifications	Geographic distribution	Strains	References
I	1a, 1b, and 1c	Africa, Asia, Australia, Europe, and the US	Pathogenic	(7, 28, 29)
II	lentogenic, mesogenic, and velogenic	Africa, Asia, North and South America, and Europe	Avirulent - low virulence	(7, 28, 29)
III		China, Japan, Taiwan, Australia, United Kingdom, Singapore and Africa	Virulent	(28, 29)
IV	–	Africa, Asia, and Europe	Pathogenic	(28)
V	4 sub-genotypes (Va-Vd)	Western Europe, Yugoslavia, Central and North America, Africa	Virulent	(7)
VI	11 sub-genotypes (VIa-VIk)	Asia, Africa, Europe, South America	Virulent	(7, 29)
VII	9 sub-genotypes (VIIa-VIIi)	Africa, China, and Europe	highly virulent	(7, 29, 30)
VIII	–	Africa and Asia	highly virulent	(7, 31)
IX	–	China	highly virulent	(29)
X	–	Argentina, Taiwan and USA	Virulent	(30)
XI	–	Madagascar	Virulent	(7)
XII	–	South America and China	Virulent	(30, 31)
XIII	3 sub-genotypes (Xia - Xia)	India, Africa, and Europe	Virulent	(7, 30)
XIV	XIVa, XIVb	Benin and Mali	Highly virulent	(7)
XV		China	Virulent	(31)
XVI	related to IV	Africa, Asia, and Europe	Virulent	(7, 29, 30)
XVII and XVIII	two sub-genotypes	Central and West Africa	highly virulent	(7)
XX	–	Tanzania and China	Virulent	(7, 29)
XXI	–	China and Nigeria	Virulent	(31)

### 2.2 Genomic structure

**Figure 1:**
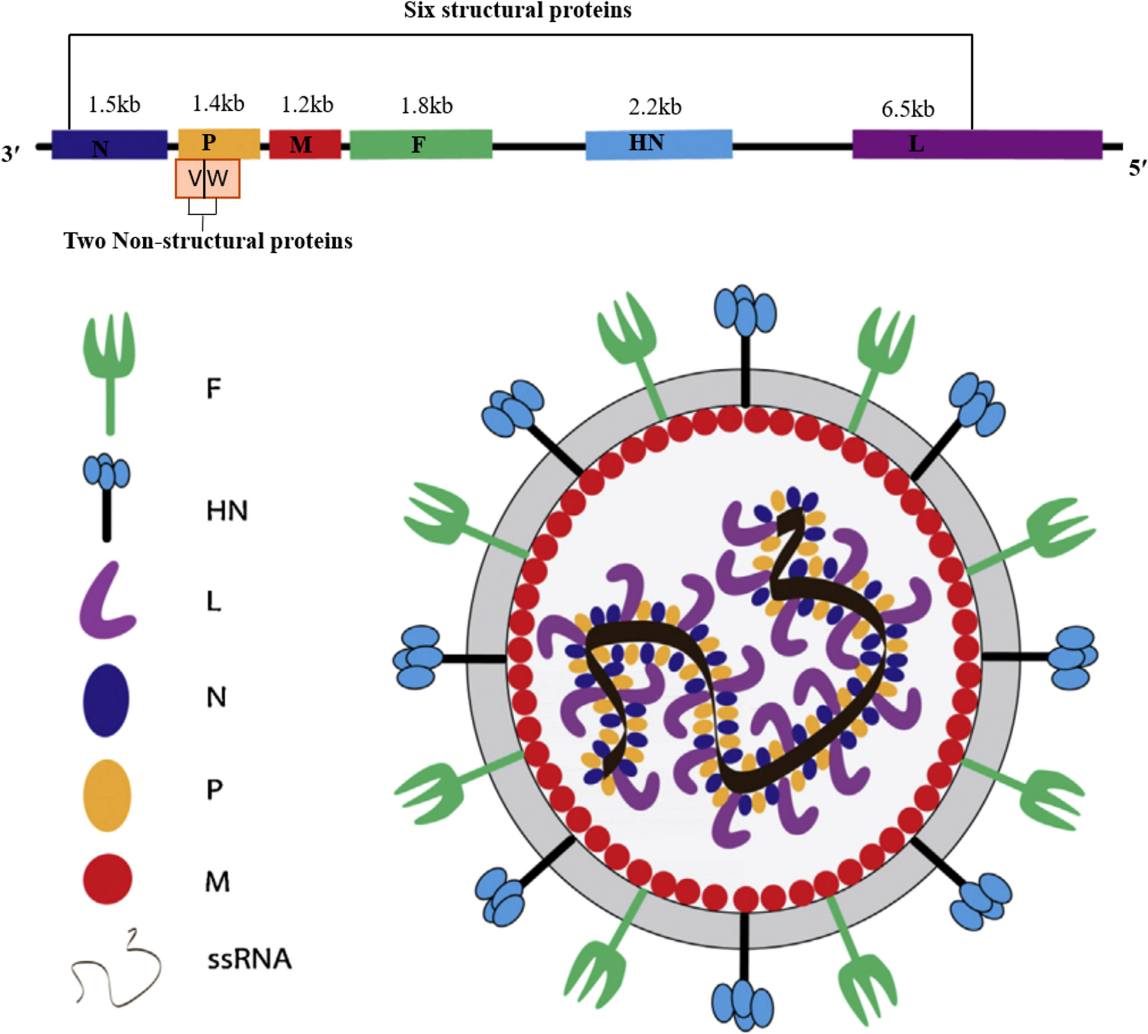
Schematic representation of the Newcastle disease virus structure, illustrating its six structural proteins: nucleoprotein (NP), phosphoprotein (P), matrix protein (M), fusion protein (F), hemagglutinin-neuraminidase (HN), and polymerase (L); its two non-structural proteins (V and W), along with the lengths of their corresponding nucleotide sequences.

The genomic structure of NDV is characterized by a single-stranded, negative-sense RNA genome of approximately 15,186, 15,192, and 15,198 nucleotides, depending on the genotype (32). The negative-sense nature of the NDV genome means that it is complementary to positive-sense mRNA and must be transcribed into positive-sense mRNA by the viral RNA-dependent polymerase before it can be translated into viral proteins within the host cells (33). As illustrated in **Figure 1**, NDV encodes six structural and two nonstructural (V and W) proteins: nucleocapsid protein (NP), phosphoprotein (P), matrix protein (M), fusion protein (F), hemagglutinin-neuraminidase (HN), and large polymerase protein (L), arranged in the order of 3’-NP-P-M-F-HN-L-5’ (4). The fusion (F) and hemagglutinin-neuraminidase (HN) glycoproteins are primary determinants of virulence, facilitating viral entry and attachment to host cells (34). The matrix (M) protein regulates viral replication and inhibits host protein synthesis, while the polymerase-associated proteins (NP, P, and L) significantly impact virulence, especially when combined, with the L protein being a major contributor (34). The P protein, essential for viral RNA synthesis, interacts with NP to form the ribonucleoprotein complex and inhibits illicit mRNA encapsidation (35).

More importantly, we also summarize the molecular structure, molecular weight, and protein length of key NDV proteins, as these factors play a crucial role in understanding viral pathogenicity and the potential for virotherapy applications (**Table 2**). The genome also features a leader (55 nucleotides) and trailer (114 nucleotides) sequence at the terminal ends (38), which play roles in viral transcription and pathogenesis. The intergenic sequences between these genes are essential for efficient transcription and have potential applications in chicken vaccine development (39, 40). NDV virulence is determined by multiple factors, including the amino acid sequence of the F protein cleavage site, tissue tropism, ability to evade the host immune response, and replication efficiency (5). The specific amino acid composition of F and the HN protein cleavage site play roles in the virulence and tissue tropism of NDV strains (5). The F protein must be cleaved by host proteases into F1 and F2 subunits for the virus to become infectious and virulent. The HN glycoprotein, which is responsible for virus attachment to host cell receptors, varies in length and is associated with pathogenicity (41). The protein contains 14 cysteine residues, 12 of which are conserved and form intramolecular disulfide linkages (37). Moreover, the M protein regulates viral replication and transcription, contributing to pathogenicity, whereas NP, P, and L proteins increase viral replication and virulence (42). In contrast, the F, M, and NP proteins are relatively conserved across NDV strains, whereas the length and molecular weight of the HN protein are more variable because of differences in the C-terminal region (39). The NP protein has been found to be highly conserved in the N-terminal region but highly variable in the C-terminal region (36). These proteins are essential for NDV virulence and pathogenesis, with the L gene identified as a key modulator of virulence (4). Furthermore, the two nonstructural proteins, W and V, are produced through RNA editing of the P gene and exhibit varying molecular weights and lengths depending on the specific strain of NDV (39). The V protein, commonly referred to as the antagonist protein, helps the virus evade the host innate immune response by facilitating escape from interferon (IFN) detection. The distribution of messenger RNA (mRNA) for these proteins is as follows: 68% for the P protein, 29% for the V protein, and 2% for the W protein (42). Although these nonstructural proteins have identical N-terminal domains (NTDs), their C-terminal domains (CTDs) differ from those of the P protein (11). The authors also noted that the V and W proteins contribute to NDV infection by antagonizing the host immune response.

**Table 2:** Molecular characteristics, including structural and nonstructural proteins, exhibit a range of molecular weights and lengths.

Classification	Proteins	Genome Length (aa)	Molecular weight (kDa)	References
Structural	NP	489	55 kDa	(36)
	P	395	50–55 kDa	(4, 37)
	M	364	40 kDa	(37)
	F	553	53 kDa	(37)
	HN	570- 616	74 kDa	(38)
	L	2204	250 kDa	(4)
Non-structural	V	239	~29–36 kDa	(37, 39)
	W	369	38 kDa	(37, 39)

aa: amino acids; kDa: kilodalton

### 2.3 Pathogenicity

The terms “pathogenicity” and “virulence” are often used interchangeably, but they have distinct meanings in microbial pathology. Pathogenicity is currently conceptualized as a qualitative assessment of a microorganism’s capacity to induce disease, encompassing variables such as infection, host entry mechanisms, proliferation, evasion of host defenses, and resultant tissue damage (43). In chickens, the pathogenicity of NDV strains varies greatly, ranging from mild respiratory or enteric disease to severe and often fatal infections (44). It depends on multiple factors, including the genetic type of the infecting virus, age, immune status, and host susceptibility (5). Botchway et al. (45) further confirmed that a virulent strain of NDV may be less lethal for a highly resistant host than for a susceptible host. Conversely, younger immunocompromised or genetically susceptible chickens are more vulnerable to severe diseases, even those caused by less virulent strains (46).

NDV is classified into different pathotypes, such as velogenic, mesogenic, and lentogenic, based on its pathogenicity (**Table 3**). Velogenic strains cause severe neurological and respiratory signs with high mortality rates and specific histopathological changes, whereas mesogenic strains cause respiratory signs with lower mortality rates (47). Lentogenic strains cause subclinical infections with mild respiratory or enteric diseases and are considered low-virulence strains (17). The vasogenic pathotype can be further classified into two subtypes: velogenic viscerotropic (VVNDV), which causes acute hemorrhagic lesions throughout the gastrointestinal tract, and velogenic neurotropic (VNNDV), which can lead to high mortality in chickens, with clinical signs ranging from respiratory distress and diarrhea to neurological disorders and sudden death (5).

**Table 3:** Classification of Newcastle disease virus pathogenicity in chickens.

Strain	Virulent	MDT	ICPI	IVPI	References
Vasogenic	High strains	< 60 hours	< 1.5	>2.5	(47)
Mesogenic	Moderately	60–90 hours	0.7-1.85	2.51	(17, 48)
Lentogenic	Low/avirulent	> 90 hours	< 0.7	<2.5	(49, 50)

MDT: mean death time; ICPI: Intracerebral pathogenicity index; IVPI: intravenous pathogenicity index.

Furthermore, the intracerebral pathogenicity index (ICPI), intravenous pathogenicity index (IVPI), and mean death time (MDT) are important measures for assessing NDV pathogenicity in chickens (6). Among these methods, ICPI is the most accurate and sensitive method for determining the virulence of NDV strains in day-old chicks, whereas IVPI is effective in six-week-old birds (47). Specifically, the ICPI values for the vaccine strains ranged from 0.0 to 0.37, indicating low virulence or non-pathogenic strains. Similarly, MDT has been used to assess the virulence of these viruses, with some strains causing death in chickens within a short period (47). Based on their MDT values, all live vaccine strains used, including the La Sota and B1 strains, were classified as lentogenic (low virulence), with the embryos remaining alive for more than 90 h (17). Studies on Brazilian commercial NDV vaccine strains in chicken clones revealed that 30 strains had an ICPI value of 0.11 and an MDT of 104 h, indicating their lentogenic (low virulence) nature (6). The author noted that the Ulster strain presented an ICPI of 0 and an MDT greater than 150 h, classifying it as lentogenic. Similarly, the Villegas-Glisson (VG/GA) strain presented an ICPI of 0.03 and an MDT of 140 h and was classified as lentogenic, indicating low virulence. The C2 strain had an ICPI of 0.04 and an MDT greater than 144 h, further confirming its lentogenic characteristics (6).

### 2.4 Insights into the NDV infection mechanism

**Figure 2:**
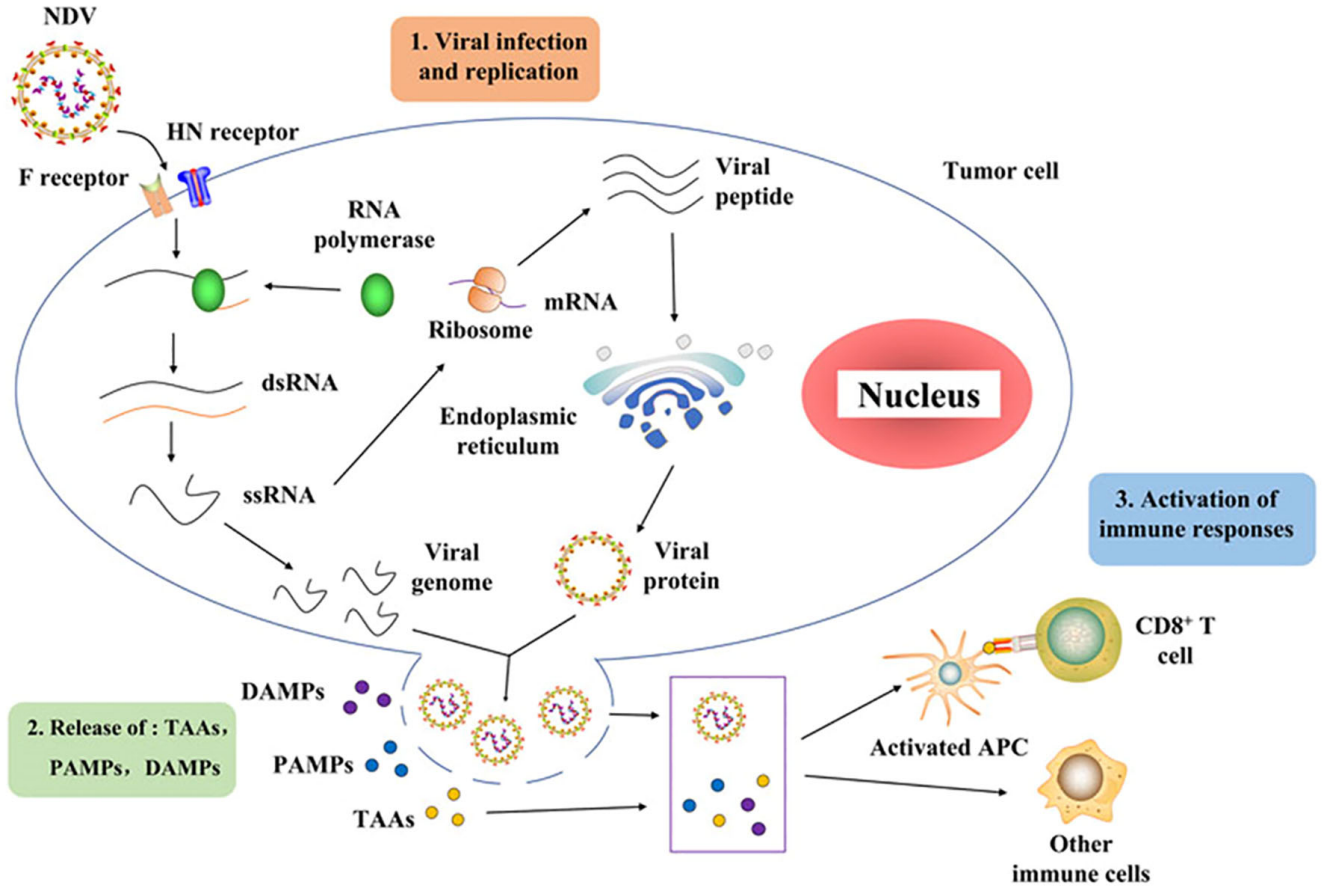
Schematic diagram of Newcastle disease virus infection cycle adapted from (52).

Recent studies have provided insights into the NDV infection mechanisms in chickens. It involves a complex interplay between molecular interactions and cellular responses (51). The initial step of NDV infection is the attachment of the virus to specific receptors on the surface of avian cells. NDVs primarily utilize sialic acid-containing glycoproteins, specifically hemagglutinin–neuraminidase (HN) and fusion (F) proteins, as receptors (42). When the HN protein binds to the receptor, structural changes lead to the breakage of the HN and F interactions. These proteins are abundantly present on the surfaces of respiratory and intestinal epithelial cells in birds (51). Moreover, the HN and F proteins play major roles in NDV virulence, as they are involved in the assembly and budding of the virus RNA genome (42). Following attachment, the viral envelope fuses with the host cell membrane, allowing the virus to enter the cell. Once inside the host cell, the RNA genome of NDV is released for replication and transcription. As depicted in **Figure 2**, NDV consists of three processes: viral infection and replication, release of TAAs, PAMPs, and DAMPs, and finally, an activated immune response. For example, viral RNA polymerase transcribes negative-sense single-stranded RNA (ssRNA) into positive-sense ssRNA, which serves as a template for translating viral proteins and replicating the genome. Newly synthesized viral proteins, such as HN and F, are processed and assembled, resulting in the budding and release of new virions that can infect other tumor cells (37, 38). During the replication cycle, the viral ssRNA genome is detected by pattern recognition receptors, such as retinoic acid-inducible gene I (RIG-I). This recognition triggers downstream signaling cascades that activate type I interferons and proinflammatory responses. As the infection progresses and tumor cells undergo lysis, they release tumor-associated antigens (TAAs), pathogen-associated molecular patterns (PAMPs), including viral proteins and RNA, and danger-associated molecular patterns (DAMPs), such as surface calreticulin, cellular DNA, and ATP (52). These molecules then activate antigen-presenting cells (APCs), such as dendritic cells, which prime CD4^+^, CD8^+^, and NK cells against tumor and viral antigens, thereby stimulating an antitumor immune response. Notably, the mechanism of NDV infection in chickens is characterized by the high virulence of the virus, which causes severe respiratory, neurological, and digestive symptoms, along with its ability to induce autophagy and inflammatory pathways that exacerbate the disease (5).

NDV primarily infects and replicates in the respiratory and digestive systems of chickens (51). Virulent strains can cause severe respiratory distress, nervous system disorders, and even sudden death. Chickens are the most susceptible domestic poultry species to NDV. NDV infection activates autophagy in chicken cells, promoting viral replication and the expression of inflammatory cytokines and chemokines, including interleukin 1 beta (IL-1β), interleukin 8 (IL-8), interleukin 18 (IL-18), C-C motif chemokine ligand 5 (CCL-5), and tumor necrosis factor alpha (TNF-α) (53). NDV-induced autophagy is positively correlated with the activation of the Caspase-1 inflammasome pathway (NLRP3) and p38/MAPK signaling pathway, resulting in increased inflammation. Finally, NDV infection can lead to mitochondrial damage and mitophagy in chicken cells, although this does not appear to significantly contribute to inflammatory responses (53).

### 2.5 NDV host genomics insights

Genomic studies have shed light on how host genetics influence immune responses to NDV infections in poultry. Host genomic insights in chickens refer to a comprehensive understanding and analysis of their genetic makeup (54), with a particular focus on the interactions between chicken DNA and various factors, including pathogens, immune responses, and other environmental or biological influences (55). In recent years, various advanced genomic approaches have been employed to identify genes associated with disease resistance, including deep RNA sequencing, whole-genome sequencing, signature of selection analysis, and genome-wide association studies (GWAS) (55). These approaches have enabled comprehensive analyses of differential gene expression, immune responses, and host-pathogen interactions in chickens. Transcriptomic studies have identified genes associated with immune regulation mechanisms and pathogen biology in chickens, including those involved in innate and adaptive immunity (56). Moreover, different NDV strains can induce varying levels of immune responses, with some strains leading to greater upregulation of avian β-defensins, cytokines, Toll-like receptors, and other immune-related genes (57). These studies provide insights into the potential genetic markers for resistance and the complex signaling pathways involved in the chicken immune response to NDV infection. Research utilizing whole-genome data from different chicken lines revealed approximately 60 variants of interest in interferon pathway genes that are known to affect susceptibility to viral pathogens (58), whereas GWAS revealed nine significant single-nucleotide polymorphisms associated with the heterophil/lymphocyte (H/L) ratio, located at 6.85 Mb on chromosome 19 (GGA19) in chickens. Furthermore, recent evidence has demonstrated that different chicken genetic lines present varying levels of resistance to NDV, which can be attributed to differences in the expression of host translation initiation factors (9, 59). For example, genes related to the integrated stress response (ISR) in chickens involve a complex interaction of eukaryotic translation initiation factor 2α (eIF2α) kinases in response to various stressors, regulating translation and cellular responses (9). The regulation of the eIF2α-mediated stress response is controlled by four key eIF2α kinases: protein kinase R (PKR), PKR-like ER kinase (PERK), general control nonderepressible 2 (GCN2), and heme-regulated inhibitor (HRI) (60). These kinases serve as central regulators of translation initiation, integrating various stress signals to reduce global protein synthesis as an adaptive response in chickens (9). The protein kinase R (PKR) pathway plays a vital role in the cellular response to viral infections and is activated upon the detection of viral components (60). Once activated, PKR phosphorylates eIF2α, leading to a decrease in global protein translation and effectively inhibiting the viral replication. In contrast, PKR, like PERK, is induced by endoplasmic reticulum (ER) stress and phosphorylates eIF2α, triggering the unfolded protein response (UPR) to alleviate ER stress (61). Similarly, the GCN2 kinase is activated by amino acid deprivation and phosphorylates eIF2α, reducing global translation while selectively upregulating stress response gene translation (9). HRI detects heme deficiency and phosphorylates eIF2α, resulting in decreased protein synthesis as an adaptive response to cellular stress (60). Through the coordinated action of these four eIF2α kinases, chickens mount an integrated stress response to a range of stimuli, including viral infection, ER stress, nutrient deprivation, and haem deficiency (60).

**Figure 3:**
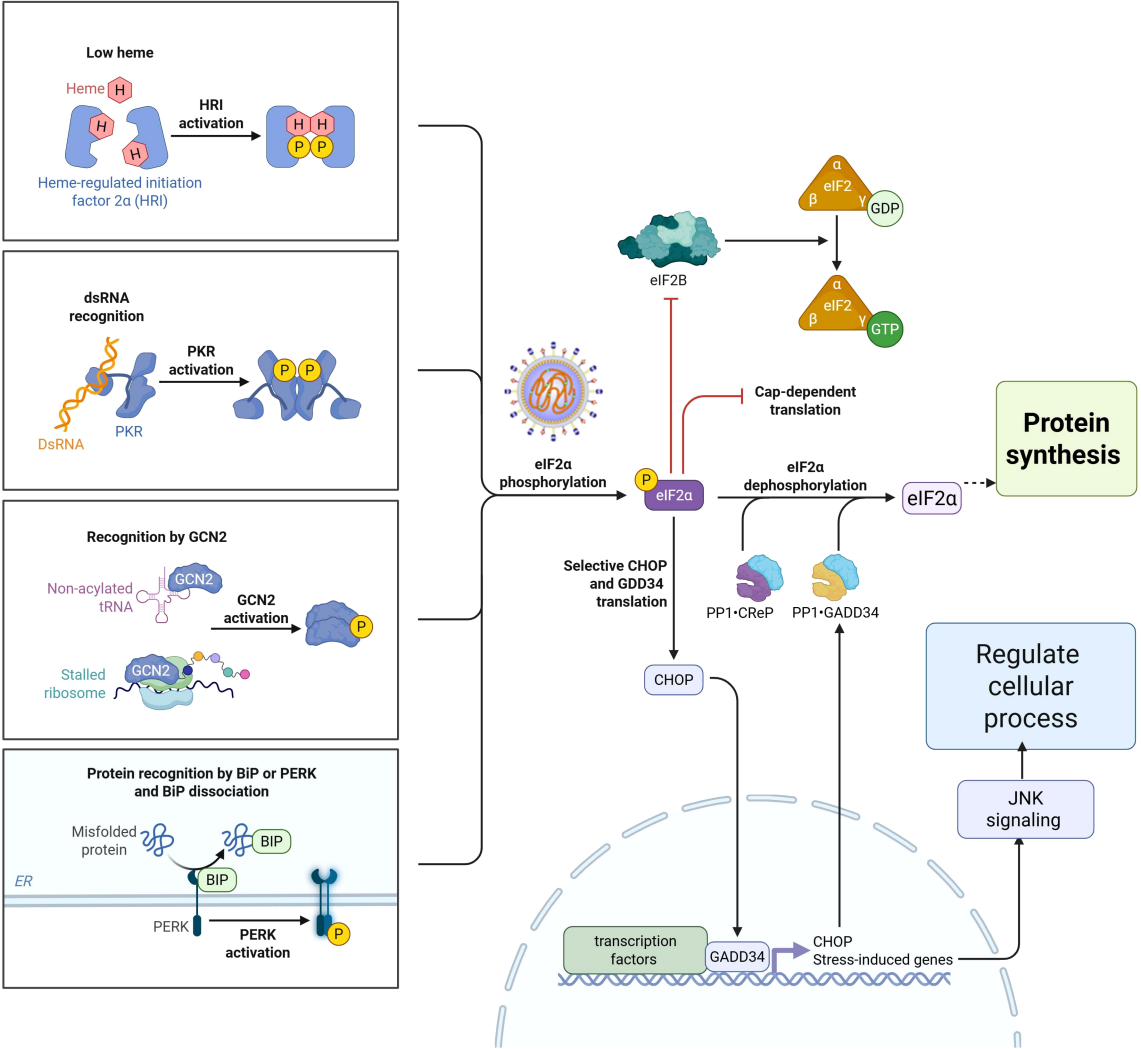
Schematic diagram of eIF2α phosphorylation signaling pathway family in response to Newcastle disease virus during protein synthesis.

Modulating eIF2α phosphorylation and the expression of eIF2 subunit genes is a vital mechanism in chickens resistant to viral infection, as it shuts down host protein synthesis and induces antiviral defenses. As illustrated in **Figure 3**, the eIF2α signaling pathway and its function are closely associated with the global attenuation of protein synthesis, with eIF2α phosphorylation serving as a key regulatory mechanism. Studies have shown that the eIF family of genes encode three subunits, eIF2α, eIF2β, and eIF2γ, produced by the EIFS1, EIFS2, and EIFS3 genes, respectively, which are essential for protein synthesis and the innate immune response in chickens (60). Phosphorylation of eIF2α following NDV infection in chicken cells leads to increased translation of activating transcription factor 4 (ATF4) and growth arrest and DNA damage-inducible 34 (GADD34), linking eIF2α phosphorylation to the immune response (60). NDV can manipulate the PKR/eIF2α signaling cascade to favor viral replication by capturing cellular mRNA during periods of stress. NDV infection downregulates the expression of EIF2B5 and EIF2S3 in chickens, potentially facilitating the shut-off of protein synthesis during the infection (60).

Furthermore, activation of the eIF2α-CHOP-BCL-2/JNK and inositol-requiring enzyme 1 alpha (IRE1α) or Jun N-terminal kinase (JNK) signaling cascades by NDV promotes apoptosis and cytokine secretion, supporting viral proliferation (55). More specifically, breed-specific responses were observed in Fayoumi chickens, which exhibited activation of eIF2 signaling and downregulation of collagen-related genes (60). Collectively, these findings highlight the importance of eIF2α in protein synthesis during the innate immune response to NDV in chickens. NDV infection in chickens activates various signaling pathways that influence viral replication and host immune response. The phosphatidylinositol 3-kinase (PI3K)/protein kinase B (Akt) signaling pathway is transiently activated early during infection, promoting cell survival and facilitating viral replication (55). The p38 MAPK pathway may regulate the expression of avian β-defensin 2 (AvBD2), an important innate immune response to NDV (57).

Investigating the molecular mechanisms underlying the host immune response to NDV is crucial for improving disease resistance in chickens. This review presents several candidate genes and signaling pathways associated with NDV resistance in chickens (**Table 4**). For example, genes related to the major histocompatibility complex (MHC-B) locus, such as LEI0070 and ADL0146, play vital roles in modulating immune responses (66). The LEI0070 marker has been associated with resistance to various pathogens and may influence antibody responses to NDV (66). The genes encoding type I interferons (IFN-α), DEAD box helicase 1 (DDX-1), interferon-γ (IFN-γ), and interleukin 6 (IL-6) are integral to the antiviral defense mechanisms in chickens infected with NDV (67). In this regard, type I interferons, particularly interferon-alpha (IFN-α), are essential for the initial defense mechanisms against NDV infections. Upon NDV infection, pattern recognition receptors (PRRs), notably RIG-I, identify viral RNA, thereby activating the RIG-I/MAVS signaling pathway, leading to IFN-α production. This pathway subsequently binds to interferon receptors on adjacent cells, activating the JAK-STAT pathway. This activation induces the expression of interferon-stimulated genes (ISGs), which produce antiviral proteins that impede viral replication and enhance the immune response, thereby establishing a robust antiviral state in host cells (68). Candidate genes, such as DDX-1, enhance the immune response to NDV; however, excessive activation can result in excessive inflammation, potentially causing tissue damage and contributing to immune pathology during severe infections. IFN-γ is crucial for robust cellular immunity and viral clearance; however, excessive production can cause tissue damage, particularly in the lungs and other organs due to heightened inflammation (5). IL-6 gene helps in the activation of adaptive immunity and infection resolution; however, excessive production can lead to detrimental effects, including a cytokine storm that causes severe inflammation, tissue damage, and potentially fatal outcomes (5).

**Table 4:** Candidate genes and genomic regions associated with the Newcastle disease virus in chickens.

Gene name	Description	Pathway	Chr	Positions	Study type	Reference
*CAMK1d*	calcium/calmodulin-dependent	cAMP-mediated signaling	1	6924097-7148087	GWAS	(62)
*NOS2*	Nitric oxide synthase	interleukins	19	9342028-9361427	GWAS	(62)
*IL8L1*	Interleukin-8	interleukins	4	50925077-50928369	GWAS	(59)
*SOCS1*	Suppressor Of Cytokine Signaling	Jak-STAT signaling	14	9341929-9345067	NGS	(63)
*CCDC3*	Coiled-coil domain-containing	negative regulation	1	7159403-7200482	GWAS	(62)
*IRF1*	Interferon Regulatory Factor	type I interferon signaling	33	17591206-17597538	RNA seq	(64)
*TIRAP*	TIR domain-containing adaptor protein	Toll-like receptor (TLR) signaling	24	447042-450173	GWAS	(65)
*KIRREL3*	a protein known as Kin of IRRE-like protein	cell adhesion	24	483929-720598	GWAS	(65)

Chr; chromosome; GWAS: genome-wide association studies.

Other host genes, including CDC16, ZBED1, MX1, and GRAP2, are also located on chromosome 1 and are strongly associated with the chicken immune response (69). CDC16 interacts with viral proteins, influencing both viral replication and immune responses. ZBED1 enhances the transcription of genes that activate innate immune responses, including cytokines and interferons (69). Conversely, insufficient expression or viral antagonism by MX1 may lead to increased viral replication and aggravated infection (70). The GRAP2 gene modulates immune responses by regulating immune cell activation and promoting cytokine production (69). Although CAMK1D affects calcium signaling, immune responses, and cell survival, CCDC3 is crucial for efficient viral entry and spread because of its involvement in cellular trafficking and cytoskeletal dynamics (69). Furthermore, TIRAP, ETS1, and KIRREL3 are important in modulating the immune response to NDV infection, influencing both viral replication and the host’s ability to control the infection (70). The same author noted that infection with Newcastle disease virus (NDV) stimulates the recognition of viral components by host cells, particularly the recognition of RNA by Toll-like receptor 3 (TLR3), which subsequently activates the TIRAP-dependent signaling pathway. Furthermore, Zhang et al. (71) identified genes such as IFIT5 and SPRY1 that were strongly associated with NDV resistance.

### 2.6 Virus diagnostic methods

**Figure 4:**
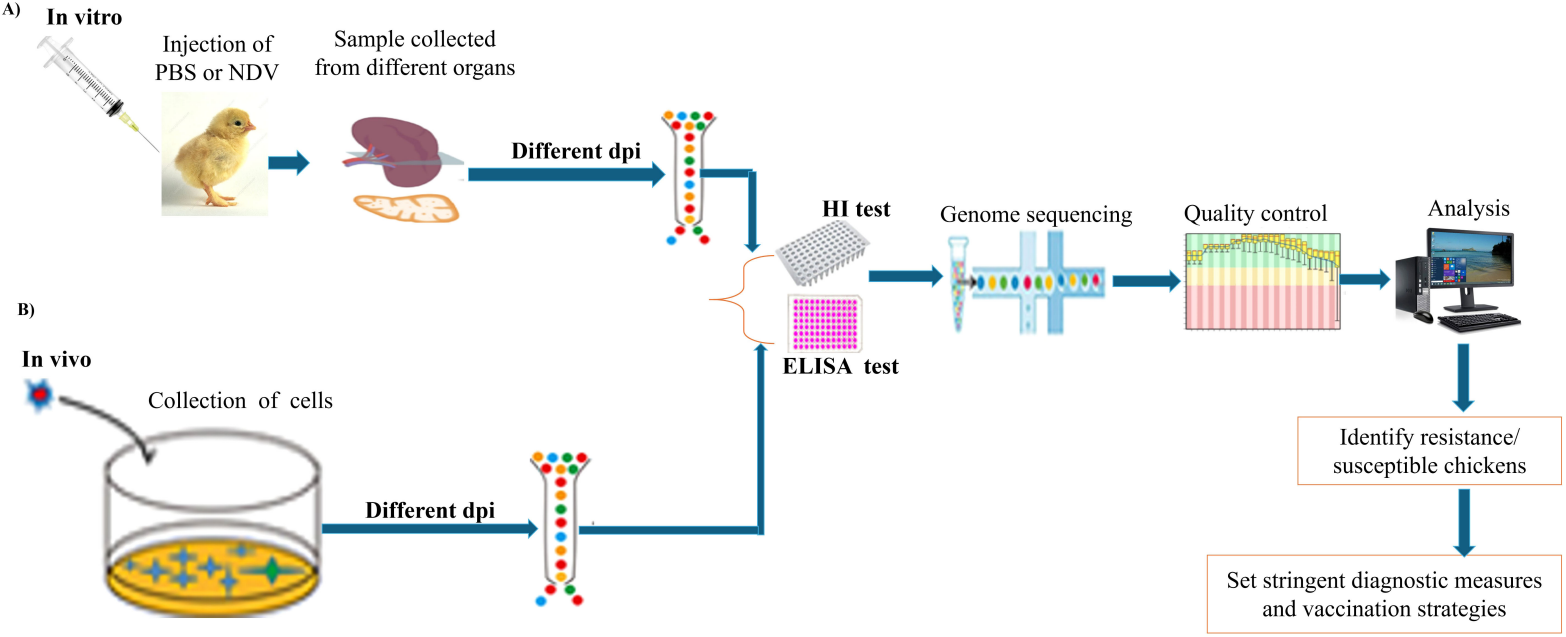
Schematic diagram of the experimental workflow for NDV diagnostic testing using different chicken organs. **(A)** *In vitro* procedure and key steps. **(B)** *In vivo* procedure and key steps.

In the poultry industry, the demand for rapid and accurate diagnostic methods for NDV is vital because of its potential to cause extensive economic losses (72). Effective diagnostics not only facilitate timely intervention but also support broader biosecurity measures that are essential for maintaining global poultry health. As a result, recent research has emphasized the importance of proper sample collection from various organs, including tracheal and cloacal swabs of live birds, and from tissues such as the spleen, lungs, intestines, liver, kidneys, heart, thymus, and brain, which are commonly used for detecting NDV in birds (73) and are commonly used for detecting NDV in birds, as illustrated (**Figure 4**). These repetitive organs are the most used organs by various researchers to detect NDV infection in birds, utilizing both *in vivo* and *in vitro* methods for subsequent sample processing, library construction, and sequencing. Tracheal samples presented the highest detection rate (92.94%), followed by spleen (90.62%) and lung (86.95%) samples (73). Wang et al. (74) performed a transcriptome analysis of spleen and thymus tissue samples obtained from NDV-infected chickens and, revealing significant alterations in gene expression associated with various cellular and biological processes. Chellappa et al. (75) conducted *in vivo* studies to assess the immune response in chickens, comparing groups that received vaccination to control groups. Their findings revealed a significant reduction in antibody titers over time in vaccinated birds as compared to unvaccinated controls, suggests that the immune response may undergo modifications as time progresses, possibly reflecting a natural waning of immunity or changes in how the chicken immune systems respond to the vaccine. Another investigation utilizing single-cell transcriptome analysis of NDV in chickens, conducted both *in vitro* and *in vivo*, revealed that the Toll-like receptor signaling pathway was the primary pathway activated following viral infection (76).

The use of optimal tissue samples plays a crucial role in gaining insights into both pathogenesis and immune responses in chickens (11). The type of tissue selected is key to ensuring an accurate diagnosis of avian diseases. Additionally, the diagnostic process must carefully consider factors such as the selection of diagnostic tests, appropriate sample types, and choice of animals for testing, as multiple tests may be necessary for proper interpretation (77). Clinical examination, which remains essential in the diagnostic workflow, continues to guide both the selection of laboratory tests and the interpretation of the results (77).

Several detection methods have been employed for NDV, including virus isolation and molecular diagnostic techniques such as RT-PCR, serotyping, and sequencing, which have become promising alternatives (11). Real-time RT-PCR offers several advantages, including high sensitivity, specificity, rapid result turnaround, and applicability to a range of sample types for avian virus detection (78). This method surpasses the hemagglutination inhibition (HI) test in terms of sensitivity and allows for further sequencing to assess viral pathotyping (78). For the accurate detection and characterization of NDV isolates, a comprehensive diagnostic approach is recommended, which combines traditional methods, such as histopathology and immunohistochemistry, with molecular techniques (72). Early detection is essential for prompt treatment and implementation of effective control measures (11). Additionally, novel approaches, such as the coagglutination kit, present promising options for rapid, cost-effective diagnosis in field settings (79).

Serological assays, such as hemagglutination (HA) and HI tests or enzyme-linked immunosorbent assays (ELISA), are commonly used for the initial detection and characterization of NDV and its related antibodies (11). HI tests are particularly favored in diagnostic laboratories because of their simplicity, rapid results, and high sensitivity and specificity. This test quantifies the titer of specific antibodies in the serum, playing a critical role in NDV surveillance, vaccine development, and, in some cases, diagnosis. ELISA is another widely used method that can detect antibodies earlier than HI tests, often identifying antibodies as early as two days post-infection. ELISA results have been shown to correlate with protective immunity against NDV challenges in chickens (11). Furthermore, the competitive ELISA format has proven effective for detecting NDV antibodies in ducks, enhancing its utility across various avian species. These serological assays are effective in detecting NDV-specific antibodies in chicken sera, making them valuable tools for monitoring infection prevalence and assessing vaccine efficacy (11).

## 3 NDV disease resistance mechanisms

Disease resistance is a critical economic trait in farm animals, as it not only protects the animals but also ensures better returns on investment for farmers. Disease resistance refers to an animal’s ability to inhibit the growth, productivity, and spread of invading pathogens within its body (80). In chickens, it is defined as the ability of an individual to disrupt the pathogen life cycle, leading to decreased pathogen load and increased antibody titters. Disease resistance in chickens is mainly influenced by genetic factors, environmental stressors, and the dynamics of their immune response (80). For example, individual birds with genetic susceptibility to diseases may have a strong immune response. However, adverse environmental conditions can increase the risk of diseases. Genetic factors play crucial roles in shaping the immune response and determining how effectively a bird can combat pathogens. Therefore, understanding the mechanisms of resistance to virulent NDV pathogens in chickens is essential for developing effective breeding strategies. Gul et al. (80) characterized disease resistance in chickens as a multifaceted interaction among these three components, as detailed below.

### 3.1 Genetic factors

Research has indicated that genetic factors significantly contribute to the resistance of chickens to NDV. Genetic differences between chicken lines with varying levels of susceptibility are key to elucidating the mechanisms underlying the immune response and identifying potential genetic markers for selecting birds with increased resistance to NDV infection (81). A study estimating the breeding value of the antibody response to NDV (Ab-NDV) via pedigree-based and genomic prediction models estimated heritability for Ab-NDV, with a genetic correlation of 0.438 between the models (58). The author further estimated the heritability of the single-trait and multiple-trait models to be 0.478 and 0.487, respectively, whereas the accuracy of genomic prediction increased from 0.086 to 0.237. In chickens, the MHC-B region contains approximately 46 genes and is associated with genetic resistance or susceptibility to infectious diseases (82). In contrast, genetic variation in chicken populations plays a role in natural resistance to specific diseases, leading to increased disease resistance (83). For example, a comparative study between Fayoumi and Leghorn breed lines revealed that the Fayoumi line was more resistant to NDV than the Leghorn line, with fewer differentially expressed genes (DEGs), a lower viral load, greater viral clearance, and greater anti-NDV antibody levels (84).

### 3.2 Environmental factors

Environmental factors, such as temperature fluctuations, humidity levels, and housing conditions, weaken the immune response of chickens, increasing their susceptibility to infection and overall health (85). Hygiene and feeding practices influence the susceptibility of chickens to viral pathogens (86). For example, gut microbiota, which are influenced by environmental factors, play a role in the immune responses of chickens to infections (85). The composition of the gut microbiome is particularly affected by poor housing conditions, with cage-free environments promoting greater diversity (85, 87). For example, hens in cage systems have lower numbers of lymphocytes and higher heterophil-to-lymphocyte (H/L) ratios, indicating a compromised immune status. Changes in environmental conditions significantly influence the spread of pathogens and the incidence of disease in chicken populations (88). Various stressors, including temperature fluctuations, humidity, and housing conditions, can compromise the immune system of chickens, increasing their susceptibility to infections (87). Akinyemi and Adewole (86) highlighted the role of prior infection with avian paramyxoviruses in conferring protection against NDV, indicating the potential influence of environmental exposure on this resistance. A study on seventy-two 32-week-old DeKalb hens revealed that a warm perch system could effectively reduce cold stress during winter, helping hens maintain body temperature without excessive feed intake or weight loss, while also improving eggshell quality (89). Another recent study on 36-week-old Hy-Line Brown laying hens reported the physiological and stress responses of hens exposed to low temperatures, indicating that cold stress can lead to tissue damage and affect physiological function (90).

### 3.3 Immune response

**Figure 5:**
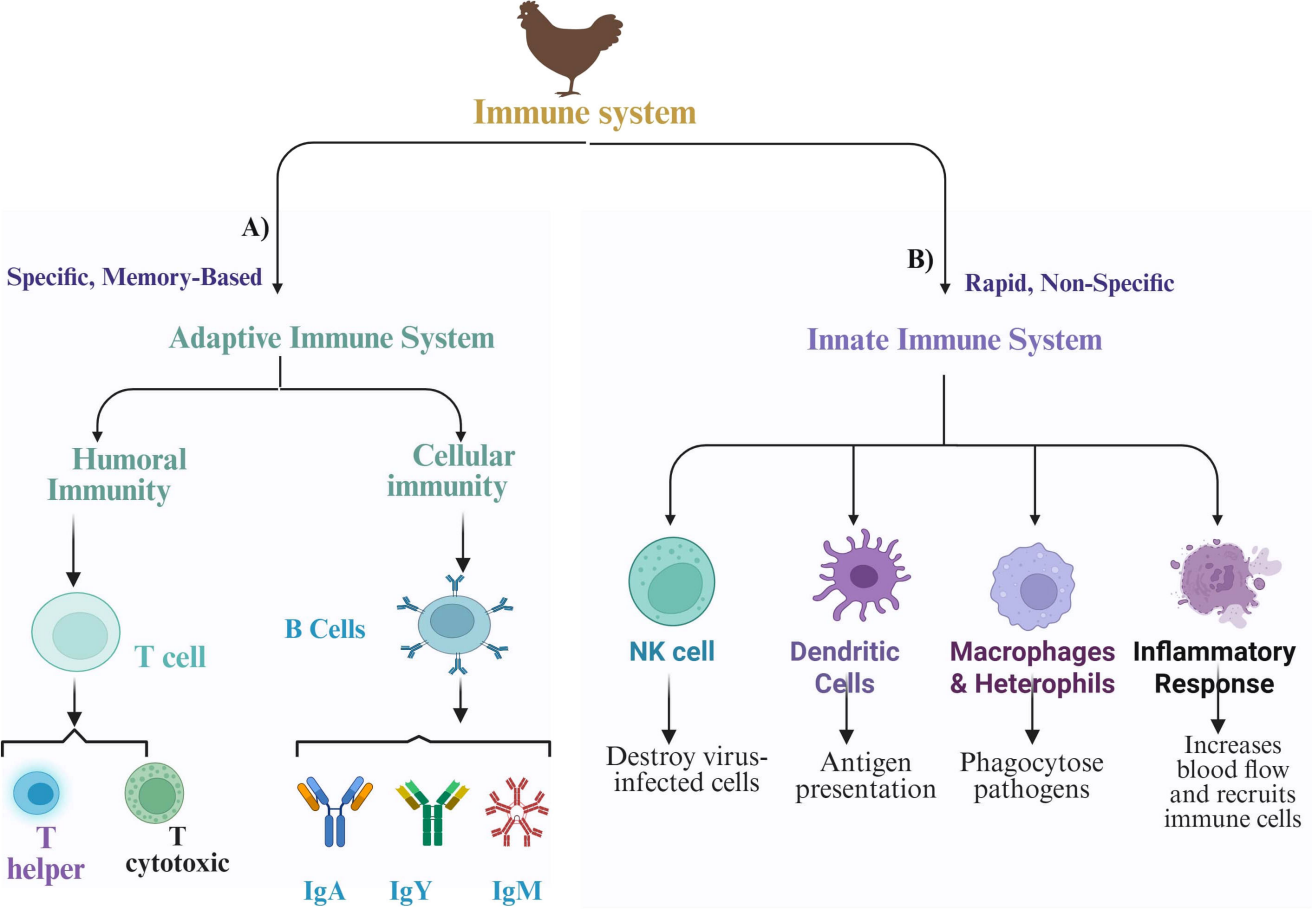
Schematic diagram of adaptive and innate immune responses against Newcastle disease virus (NDV) in chickens. **(A)** Adaptive immunity and its key components. **(B)** Innate immunity and its key components.

The immune response to NDV in chickens is a complex polygenic trait, with multiple genes contributing to genetic variations in antibody response and disease resistance (91). A greater antibody response is associated with better resistance; however, an excessively strong immune response can sometimes lead to increased susceptibility and pathology (92). As shown in **Figure 5**, the immune response comprises both innate (first-line defense) and adaptive (second-line defense) components that work together to protect birds from infection and disease (93). The innate system provides an immediate, nonspecific response to contain infections, whereas the adaptive system offers a specific, long-lasting response that can remember and more effectively combat pathogens upon re-exposure (94). Like mammals, chickens initiate their adaptive immune response through the activation of antigen-presenting cells (APCs) (94). The immune system then triggers either a humoral response (such as antibody production) or a cell-mediated response (which activates antiviral and antitumor mechanisms) (53). Both responses, driven by T and B lymphocytes, are characterized by specificity and memory, essential for effective vaccination. In chickens, the immune system consists of various organs, tissues, cells, and molecules, all working together to generate appropriate immune responses and establish immunological memory (83). The humoral immunity is primarily mediated by B cells that differentiate into plasma cells that secrete antibodies specific to the encountered antigens. In contrast, cellular immunity is driven mainly by T cells and does not rely on antibodies; instead, it involves the direct action of T cells against infected cells (94). Chickens possess a strong immune response system that combats various threats, particularly viruses, by producing antibodies (IgA, IgM, and IgY) (5, 91). These antibodies, or immunoglobulins, have a Y-shaped structure with heavy and light chains. Importantly, IgY antibodies exhibit high specificity and effectiveness in neutralizing viral infections (95). IgA protects mucosal surfaces, whereas IgM enhances early immune responses by forming pentameric structures that facilitate opsonization and complement protein activation (96).

Currently, different researchers have evaluated NDV adaptive immune responses by measuring antibody titters at different ages and time points (**Table 5**) and have observed significant differences. For example, on day 0 (hatch), chicks primarily rely on maternally derived antibodies (MDA), and the live NDV LaSota strain vaccine does not substantially stimulate adaptive immunity at this stage (100). Within the first 1–2 weeks post-vaccination, the replicating live attenuated NDV triggers an innate immune response at the site of administration (93). Both humoral and cellular immune responses can be activated within 2–4 weeks following vaccination (100), with B cells producing NDV-specific antibodies and T cells differentiating into effector and memory T cells. By 4–6 weeks, strong antibody titers along with established populations of memory B and T cells provide long-term immune memory against the target antigen expressed by the NDV vector (101). At4 weeks of age, chicken MDA levels decrease below protective thresholds and then significantly increase at 7 and 14 days after vaccination with either the virulent ZG1999HDS strain or the lentogenic LaSota vaccine strain (98).

**Table 5:** Antibody titters of the adaptive immune response to Newcastle disease virus at different ages and time points.

Age (in Days)	Descriptions	Types of Immune	References
5-7	Stimulation and induction of antibody production by antigens to initiate the humoral immune response	Humoral Immunity	(76, 97);
7-14	Detectable levels of antibodies in response to vaccination		
14-21	Antibody levels continue to rise as B cells produce more antibodies		
21-42	Peak antibody titer response is typically observed		
42-72	Antibody levels start to decline but remain protective		
5-7	Activation of innate immune cells like macrophages and dendritic cells	Cellular Immunity	(76, 97 98)
7-14	Proliferation of T cells, including CD4^+^ helper T cells and CD8^+^ cytotoxic T cells		
14-21	Activated T cells produce increased levels of cytokines like IFN-γ, IL-2, and IL-4		
21-42	Peaks with high levels of activated T cells and cytokine production		
42-72	Persistence of memory T cells		
72-91	Immune mechanisms, including memory B and T cells, provide long-lasting protection	Both are established	(97, 99)

Moreover, studies have demonstrated that chicken immune development exhibits three distinct patterns: Down-Up, Up-Down, and Up-Up (102). These patterns are characterized by fluctuations in cytokine levels and immune cell function, with the immune system not fully maturing until a later stage. The down-up, down-up pattern is characterized by an initial decrease in cytokine levels and certain immune indicators, followed by a subsequent increase. Notably, the lowest levels of immune function were observed between days 6 and 13 (102). In the up-down pattern, the highest levels of various immune components were observed between days 30 and 34. These include nonspecific cellular immunity components, such as the ratio of peripheral blood mononuclear macrophages; specific cellular immunity components, such as the ratio of peripheral blood helper T cells and the proliferative activity of T and B cells; and mucosal immunity components, including ileal CD4, TGF-β1, and IgA mRNA levels (102). In the Up-Up pattern, various immune components increased from days 1 to 34. Nonspecific cellular immunity components, including serum nitric oxide, C3, and C4, were elevated, as were specific cellular immunity components, such as the spleen index, peripheral blood IL-2, IFN-γ/IL-4 ratio, cytotoxic T-cell ratio, and splenic NF-κB mRNA. Additionally, humoral immunity is enhanced, as evidenced by increased serum IgG levels, whereas the levels of mucosal immunity components, including ileal MHC-II and pIgR mRNAs and ileal mucosal IgA, also increase during this period (102).

Furthermore, the immune response to NDV is characterized by the expression of various cytokines. For example, infection with virulent strains of NDV results in elevated levels of interleukin-1β (IL-1β), which is a crucial mediator of inflammation (32). Research has demonstrated that the expression of IL-1β is regulated by the NLRP3/caspase-1 pathway during NDV infection, highlighting the importance of specific immune pathways in determining disease outcomes (32). The expression patterns of cytokines, such as type I interferons (IFN-α), interferon-γ (IFN-γ), and interleukin 6 (IL-6), in relation to the NDV strain and the timing of the immune response underline the importance of both genetic factors and the timing of immune activation (103). Highly virulent NDV strains are known to elicit rapid and robust expression of proinflammatory cytokines, particularly IL-1β and IL-6, in the lymphoid tissues of young chicks (104). The expression of IFN-γ is also upregulated, although to a lesser extent (104).

## 4 Impact of NDV on chicken immune performance

Newcastle disease virus infection can significantly impact chicken immune performance and health. Numerous studies have elucidated the mechanisms by which NDV influences immune responses and the efficacy of vaccination (105). NDV infection can negatively affect chicken performance by decreasing body weight gain and efficiency, particularly during the early growth stages (106, 107). The virus causes severe pancreatic damage, which reduces enzyme activity and expression, potentially contributing to impaired growth performance (106). One of the primary mechanisms by which NDV affects chicken performance is through its influence on the immune system. Infected chickens present elevated levels of corticosterone, a stress hormone that is associated with decreased growth rates, compromised antibody responses, and lower lymphocyte counts (106). This hormonal response may negatively impact the overall health of chickens, increasing their susceptibility to further infections and diseases, which in turn exacerbates production losses (106). Furthermore, NDV infection has been shown to disrupt the composition of the gut microbiota, which is essential for nutrient absorption and overall health. Such disruption of the gut microbiota can lead to impaired growth performance and increased susceptibility to systemic diseases (108). This relationship between NDV and the gut microbiota highlights the interaction between viral infections and the physiological state of the host.

Additionally, NDV has been shown to replicate effectively within chicken macrophages, which are key players in the immune system. This replication may result in the polarized activation of these immune cells, potentially altering the immune response and diminishing the chickens’ ability to fight off other pathogens (109). By manipulating immune responses, NDV not only ensures its own survival but also compromises host health, leading to reduced productivity and increased mortality rates in severe cases (71). The economic implications of NDV are profound, particularly in regions where poultry production is vital for income and nutrition. This disease can cause mass mortality and substantial declines in productivity, adversely affecting both meat and egg production (110). In many developing countries, the impact of NDV is particularly severe due to inadequate vaccination coverage and insufficient biosecurity measures, which worsen the spread of the virus and its economic consequences (110, 111). For example, in rural communities, outbreaks of NDV can devastate local poultry populations, resulting in food insecurity and financial distress for households that depend on chicken farming for their livelihoods (110).

Research indicates that vaccination against NDV enhances the immune response in chickens, leading to improved disease resistance. The selection for high antibody responsiveness to NDV vaccination has been shown to improve natural immunity, suggesting that genetic selection can increase disease tolerance in chickens. Vaccination-induced stress can increase serum stress hormones, affect growth performance, and trigger lymphocyte apoptosis, with optimal antibody titers achieved at lower doses (112). Lower doses of the NDV vaccine (2–6 doses) were found to be optimal, producing peak antibody titers without compromising growth performance (112), whereas higher doses led to increased lymphocyte apoptosis and reduced feed efficiency. Moreover, combining live and inactivated NDV vaccines provides superior protection compared with live vaccines alone (105).

Recombinant NDV vaccines have demonstrated effectiveness against NDV and other avian pathogens, enhancing overall chicken flock productivity (53). Various administration routes for NDV vaccines, such as oculonasal or nebulization, have been evaluated for their immunogenicity, indicating that these methods can effectively stimulate the immune system in day-old chicks. Innovative vaccine formulations, including those encapsulated in chitosan nanoparticles, have shown the capacity to induce effective immune responses while maintaining stability and efficacy (25). The immune response to NDV vaccination can be compromised by concurrent infections, such as chicken infectious anemia virus (CIAV), which causes immunosuppression and affects bird performance (105). These advancements in vaccine technology are essential for ensuring that chickens effectively withstand NDV challenges. However, the decline in these antibodies within several weeks can render chicks susceptible to infection if they are not adequately vaccinated (98). Consequently, the timing and effectiveness of vaccination programs are critical for managing NDV outbreaks and ensuring optimal performance in poultry production.

## 5 Conclusions

In this review, we have explored the molecular mechanisms of NDV, including its virulence determinants, infection routes, host resistance factors, and immune evasion strategies, as well as the key candidate genes and genomic regions involved in chickens. We also highlight the interaction between genetic factors, environmental influences, and immune responses that shape NDV resistance. While significant progress has been made in understanding the interaction of host immunity and cytokine regulation, NDV’s ability to evade immune defense remains a challenge. The virus significantly impacts the immune performance of chickens, often causing immunosuppression in severe cases and compromising overall poultry health and productivity. Understanding these interactions is essential for developing effective control strategies against this economically important pathogen. Consequently, a multidisciplinary approach combining genomics, immunology, and epidemiology will be key to will provide new insights into the replication and pathogenesis of NDV. Further research should focus on elucidating host-pathogen interactions at the molecular level, optimizing immune modulation strategies, and developing sustainable solutions tailored to diverse poultry production systems. This comprehensive overview underscores the advancements in understanding the molecular basis of NDV and the potential for developing immunological-based techniques for targeted disease control measures in the poultry industry.
